# Qualitative and Quantitative NAD^+^ Metabolomics Lead to Discovery of Multiple Functional Nicotinate *N*-Glycosyltransferase in *Arabidopsis*

**DOI:** 10.3389/fpls.2019.01164

**Published:** 2019-09-27

**Authors:** Lingyun Liu, Fengxia Zhang, Guosheng Li, Guodong Wang

**Affiliations:** ^1^State Key Laboratory of Plant Genomics and National Center for Plant Gene Research (Beijing), Institute of Genetics and Developmental Biology, The Innovative Academy of Seed Design, Chinese Academy of Sciences, Beijing, China; ^2^College of Advanced Agricultural Sciences, University of the Chinese Academy of Sciences, Beijing, China

**Keywords:** NAD, nicotinate, *N*-glycosyltransferase, Preiss-Handler pathway, *Arabidopsis*

## Abstract

The Preiss-Handler pathway, which salvages nicotinate (NA) for NAD synthesis, is a conserved biochemical pathway in land plants. We previously demonstrated that various NA conjugations (mainly methylation and glycosylation) shared the NA detoxification function in all tested plants. It remains unclear whether other NA conjugates with low abundance exist in plants. In this study, we discovered at least two additional NA *N*-glycosides in *Arabidopsis*, which was tentatively elucidated as nicotinate *N*-pentoside (Na*N*P) and NA *N*-rhamoside (Na*N*Rha), using liquid chromatography triple-quadrupole mass spectrometry (LC-QQQ-MS) with precursor ion-scanning (PreIS). We further quantitatively profile the NAD^+^-related metabolites in 24 tissues of *Arabidopsis*. Biochemical assays of UGT76C family revealed that UGT76C5 (encoded by *At5g05890*, previously identified as Na*N*GT) was a multiple functional nicotinate *N*-glycosyltransferase, with high preference to UDP-xylose and UDP-arabinose. The deficiency of Na*N*P and Na*N*Rha in *ugt76c5* mutant suggested that UGT76C5 is responsible for biosynthesis of Na*N*P and Na*N*Rha *in planta*. We also identify one amino acid difference in PSPG (plant secondary product glycosyltransferase) motif is responsible for the divergence of Na*N*GT (UGT76C4) and UGT76C5. Taken together, our study not only identifies a novel nicotinate *N*-glycosyltransferase but also paves the way for investigations of the *in planta* physiological functions of various NA conjugations.

## Introduction

**Figure 1:**
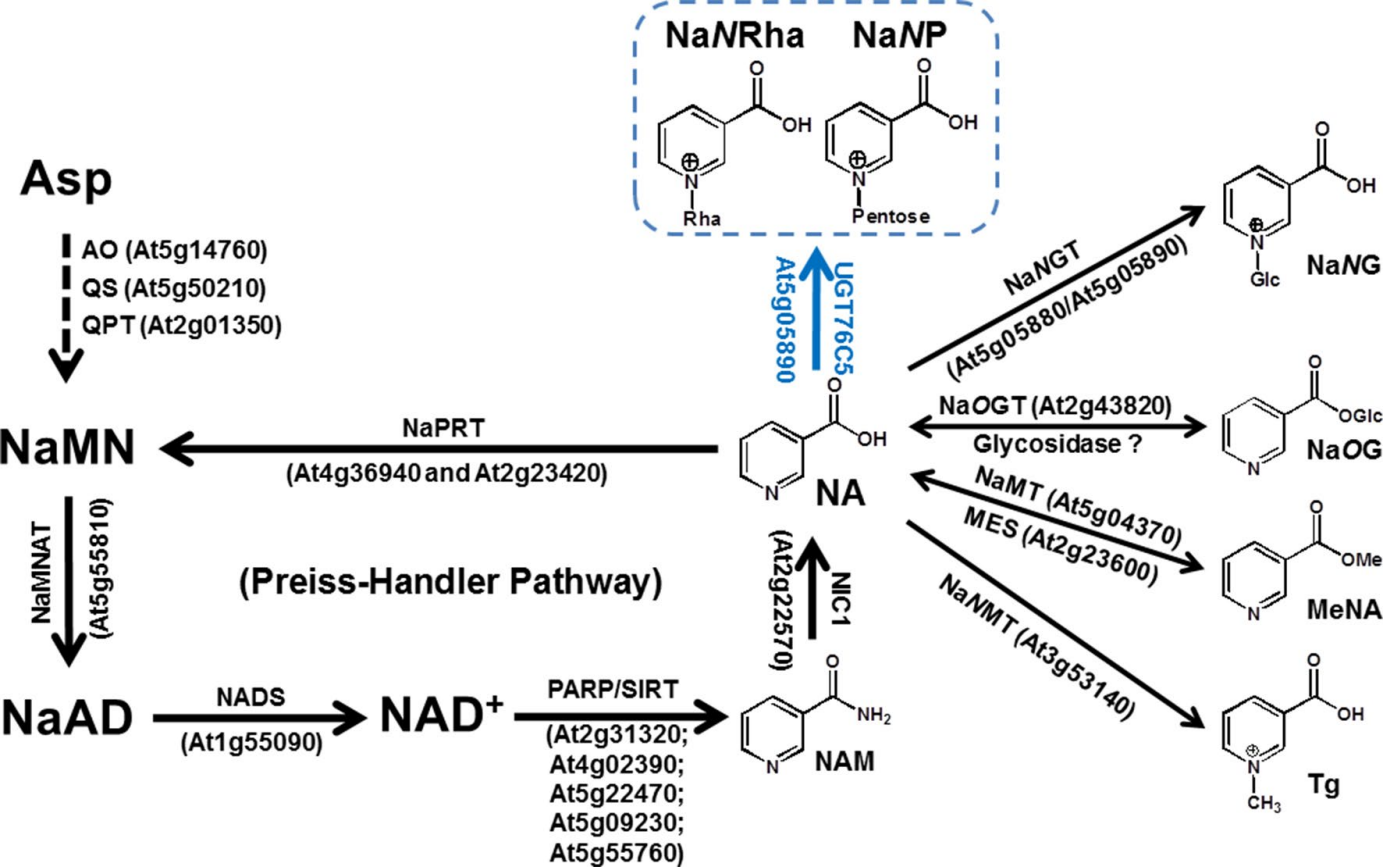
NAD Metabolism and Nicotinate Conjugations in *Arabidopsis thaliana*. The reaction catalyzed by UGT76C5 and its products, Na*N*P (nicotinate *N*-pentoside) and Na*N*Rha (nicotinate *N*-rhamnose), are highlighted. The genes, which encode characterized enzymes in *Arabidopsis*, are also indicated. Abbreviations: AO, aspartate oxidase; MeNA, methyl nicotinate; MES, methylesterase; NA, nicotinate; NaAD, nicotinate adenine dinucleotide; NAD, nicotinamide adenine dinucleotide; NADS, NAD synthase; NAM, nicotinamide; NaMN, nicotinate mononucleotide; NaMNAT, NaMN adenylyltransferase; NaMT, nicotinate methyltransferase; Na*N*MT, nicotinate *N*-methyltransferase; Na*O*G, nicotinate *O*-glucoside; Na*O*GT, nicotinate *O*-glucosyltransferase; Na*N*G, nicotinate *N*-glucoside; Na*N*GT, nicotinate *N*-glucosyltransferase; NaPRT, nicotinate phosphoribosyltransferase; NIC1, nictinamidase 1; PARP, poly(ADP-ribose) polymerase; QPT, quinolinate phosphoribosyltransferase; QS, quinolinate synthase; SIRT, sirtuins (NAD dependent protein deacetylases); Tg, trigonelline.

It has long been known that NAD is a coenzyme that serves as an electron carrier in hundreds of redox reactions. In land plants, NAD can be biosynthesized *via* both a *de novo* pathway starting from aspartate (Asp) or by the Preiss-Handler salvage pathway (Katoh et al., 2006; Noctor et al., 2006; Wang and Pichersky, 2007). The Preiss-Handler pathway, shared by land plants and most bacteria, starts with nicotinate (NA), which generated by nicotinamidase (NIC1, EC 3.5.1.19, encoded by *At2g22570*) from nicotinamide (NAM). In *Arabidopsis*, NA was catalyzed sequentially by nicotinate phosphoribosyltransferase (NaPRT, EC 6.3.4.21, encoded by *At2g23420* and *At4g36940*), nicotinate mononucleotide adenylyltransferase (NaMNAT, EC 2.7.7.18, encoded by *At5g55810*), and NAD synthase (NADS, EC 6.3.5.1, encoded by *At1g55090*) (**Figure 1**) (Preiss and Handler, 1958a, Preiss and Handler, 1958b; Wang and Pichersky, 2007). Feeding experiments, typically using carbonyl [^14^C]-nicotinamide ([^14^C]-NAM) as a tracer, showed that NA in plants, in addition to being a precursor for NAD replenishment, can also be converted into a variety of NA conjugations (**Figure 1**). The over-accumulation of NA has been demonstrated to cause toxicity in plant cells (Zheng et al., 2005; Wang and Pichersky, 2007; Li et al., 2015; Li et al., 2017). We previously proposed that plant developed various strategies to deal with the NA toxicity based on the fact that various conjugates of NA (glycosylation or methylation at the *N*-position or carboxyl group of NA) had been detected in plants. We had demonstrated that NA *O*-glucosylation catalyzed by nicotinate *O*-glucosyltransferase (Na*O*GT, encoded by *At2g43820*), a process likely restricted to the *Brassicaceae*, functions to detoxify NA and involve into seed germination under stress conditions (Li et al., 2015). Two genes encoding NA *N*-glucosyltransferase (*Na*N*GT*s, EC 2.4.1.196, encoded by *At5g05880* and *At5g05890*) were also cloned and functional characterized (Li et al., 2015). However, *N*-methylnicotinate, also called trigonelline, is a ubiquitous NA conjugate in land plants. NA detoxification conferred by nicotinate *N*-methyltransferase (Na*N*MT, EC 2.1.1.7, encoded by *At3g53140*) might have facilitated the retention of the Preiss-Handler pathway in land plants (Li et al., 2017). More recently, the reversible methylation at carboxyl group of NA had been characterized as long-distance transport of NAD precursors in *Arabidopsis thaliana* (Wu et al., 2018).

Besides the above-mentioned NA conjugations, it is an open question whether additional NA conjugates exist in *Arabidopsis thaliana*. Given the low resolution of TLC analysis used in previous studies on NA metabolism, the discovery of unknown NA conjugates, especially for those which are in low abundance, is really limited. Recently, the mass spectrometry with precursor ion-scanning (PreIS) becomes an alternative choice to discover a series of derivatives with a common substructure (such as the NA moiety in NA conjugates) (Du et al., 2018). The QQQ-MS with PreIS provides much higher resolution and selectivity than the classical TLC analysis.

In this study, we first discovered two types of NA *N*-glycosides in *Arabidopsis thaliana* by using LC-QQQ-MS with PreIS; one peak was identified as NA *N*-pentoside, and another one was tentatively identified as NA *N*-rhamoside. Quantitative NAD^+^ metabolomics of 24 *Arabidopsis* tissues were determined using LC-QQQ-MS with the optimized MRM (multiple reaction monitoring) conditions. We further determine UGT76C5 (encoded by *At5g05890*) as a multiple functional NA *N*-glycosyltransferase with sugar-donor promiscuity. One amino acid in PSPG (plant secondary product glycosyltransferase) motif is responsible for the biochemical difference of UGT76C4 (Na*N*GT) and UGT76C5.

## Materials and Methods

### Plant Materials and Chemicals

The wild-type and transgenic *Arabidopsis thaliana* (Col-0 ecotype) lines were grown on soil at 22°C under a 16-h light (120 µmol m^−2^ s^−1^)/8-h dark cycle, 60% relative humidity. To harvest young seedlings, the sterilized seeds were sown on ½ MS plates (0.8% agar). The plates were incubated at 4°C for 3 days and then transferred into growth chamber with long-day condition (16-h light/8-h dark cycle, 60 µmol m^−2^ s^−1^, 60% relative humidity). All chemicals used in this study were purchased from Sigma-Aldrich (USA) except the radiolabeled (carboxyl-^14^C)-NA (55 mCi/mmol) were purchased from American Radiolabeled Chemicals (USA). UDP-xylose and UDP-arabinose were purchased from Sugars Tech company (Qingdao, China).

### LC-QQQ-MS Analysis With Precursor Ion Scan and MRM

The extraction and measurement of NA, its conjugates, and other NAD-related chemicals in *Arabidopsis* are carried out as described previously (Li et al., 2015; Wu et al., 2018). An Agilent 1290 ultra-high performance liquid chromatography coupled with an Agilent 6495 Triple Quadrupole Mass Spectrometer was applied for metabolites analysis. Samples were separated on an ACQUITY UPLC BEH Amide column (1.7 μm, 2.1 mm i.d. × 100 mm) (Waters Corporation). The mobile phase consisted of 50% acetonitrile (A) and 95% acetonitrile (B). The mobile phases A and B contained 0.15% ammonium hydroxide and 5mM ammonium acetate. A linear gradient elution program was used: 0–0.5 min, 98% B; 0.5–6 min, 98–75% B; and 6–7 min, 75–25% B. The column temperature was 35°C. The flow rate was 0.35 ml/min. Precursor ion scan mode was used for identifying the unknown NA *N*-glycosides, and MRM mode was used for the quantification of the interested metabolites. The MRM parameters were listed in **Supplemental Table 1**. The MS acquisition and precursor ion scan parameters were as follows: high-purity nitrogen (N_2_) was used as the nebulizer (12 L/min, 200°C, 30 psig) and sheath gas (12 L/min, 350°C). The parent ion ranges from *m/z* 50 to 800. The precursor ion was *m/z* 124, fragment was 380 v, and collision voltage was 20 v. Data were analyzed by MassHunter Software B07.01 (Agilent).

### Site-Directed Mutagenesis and Glycosyltransferase Assays

Mutated *UGT76C4* and *UGT76C5* were generated by using a previously described PCR-mediated method (Ho et al., 1989). The primers used are detailed in **Supplemental Table 2**.

Na*N*GT proteins, including UGT76C4, UGT76C5, and their mutants, expression in *E. coli*, purification of recombinant Na*N*GTs’ and GT assays are carried out as described previously (Li et al., 2015).

## Results and Discussion

### Novel NA Conjugates Detected in *Arabidopsis thaliana*

**Figure 2:**
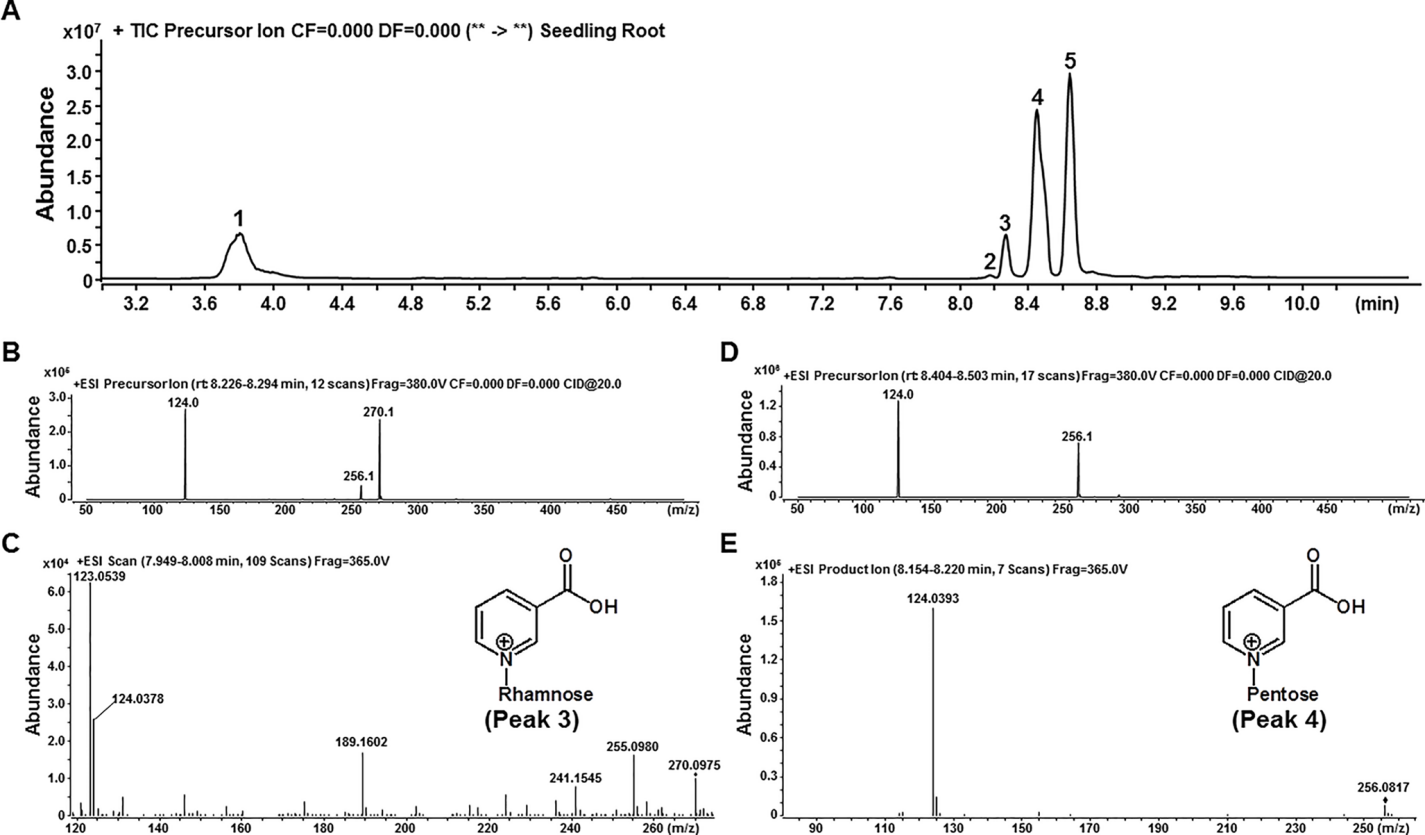
Two novel NA glycosides detected by using LC-QQQ-MS with PreIS of *m/z* 124. **(A)** Liquid chromatograph of total five different NA glycosides, which detected in the root tissue of 15-day-old *Arabidopsis* seedlings. Peaks 1, 2, and 5 were elucidated as Na*O*G, NaR (nicotinate riboside), and Na*N*G by comparison with authentic compounds. **(B** and **C)**. Mass spectra of Peak 3, which generated with QQQ-MS **(B)** and qTOF-MS **(C)**. **(D** and **E)**. Mass spectra of Peak 4, which generated with QQQ-MS **(D)** and qTOF-MS **(E)**.

Based on mass spectrometry cleavage rules of known NA conjugates (such as Na*N*GT and Na*O*GT), the *m/z* 124.0 fragment (NA moiety, C_6_H_6_NO_2_^+^, the calculated *m/z* 124.0398) was thus chosen as signature ion for PreIS to screen unknown NA conjugates (Li et al., 2015). We firstly applied LC-QQQ-MS with PreIS of *m/z* 124 to detect unknown NA conjugates from the root of 15-day-old *Arabidopsis* seedlings. As shown in **Figure 2A**, five compounds probably with NA moiety were detected on the chromatogram. Among these five putative NA conjugates, peaks 1, 2, and 5 were elucidated as Na*O*G, NaR (nicotinate riboside), and Na*N*G by comparison with authentic compounds. The chemical structures of peaks 3 and 4 were further elucidated by product ion mode. The [M+H]^+^ ions for peaks 3 and 4 display monoisotopic *m/z* at 270.1 (270.0975 on qTOF-MS) and 256.1 (256.0817 on qTOF-MS), respectively. The lost neutral fragment for peak 3 has a predicted molecular weight of 146.0 (C_6_H_10_O_4_) consistent with a rhamnose moiety (**Figure 2B–C**). However, the lost neutral fragment for peak 4 has a predicted molecular weight of 132.0 (C_5_H_8_O_4_), consistent with a pentose moiety (**Figure 2D–E**). Therefore, peaks 3 and 4 were tentatively elucidated as nicotinate *N*-rhamnoside and nicotinate *N*-pentoside, respectively, based on the polarity of their chemical structures and their behavior in our HPLC analytical system (**Figure 2A**). The pentose moiety attached to nicotinate could be either xylose or arabinose, both of which shared the same chemical formula (C_5_H_10_O_5_) and commonly detected in plants (Reiter, 2008; Caputi et al., 2012). It is noteworthy that NA *N*-arabinoside and NA *N*-xyloside could not be well separated on our LC-QQQ-MS system (**Supplemental Figure 1**); we thus use NA *N*-pentoside (Na*N*P) to express the sum of NA *N*-arabinoside and NA *N*-xyloside for further analysis.

### Tissue Specificity of NAD^+^ Metabolomics

**Figure 3:**
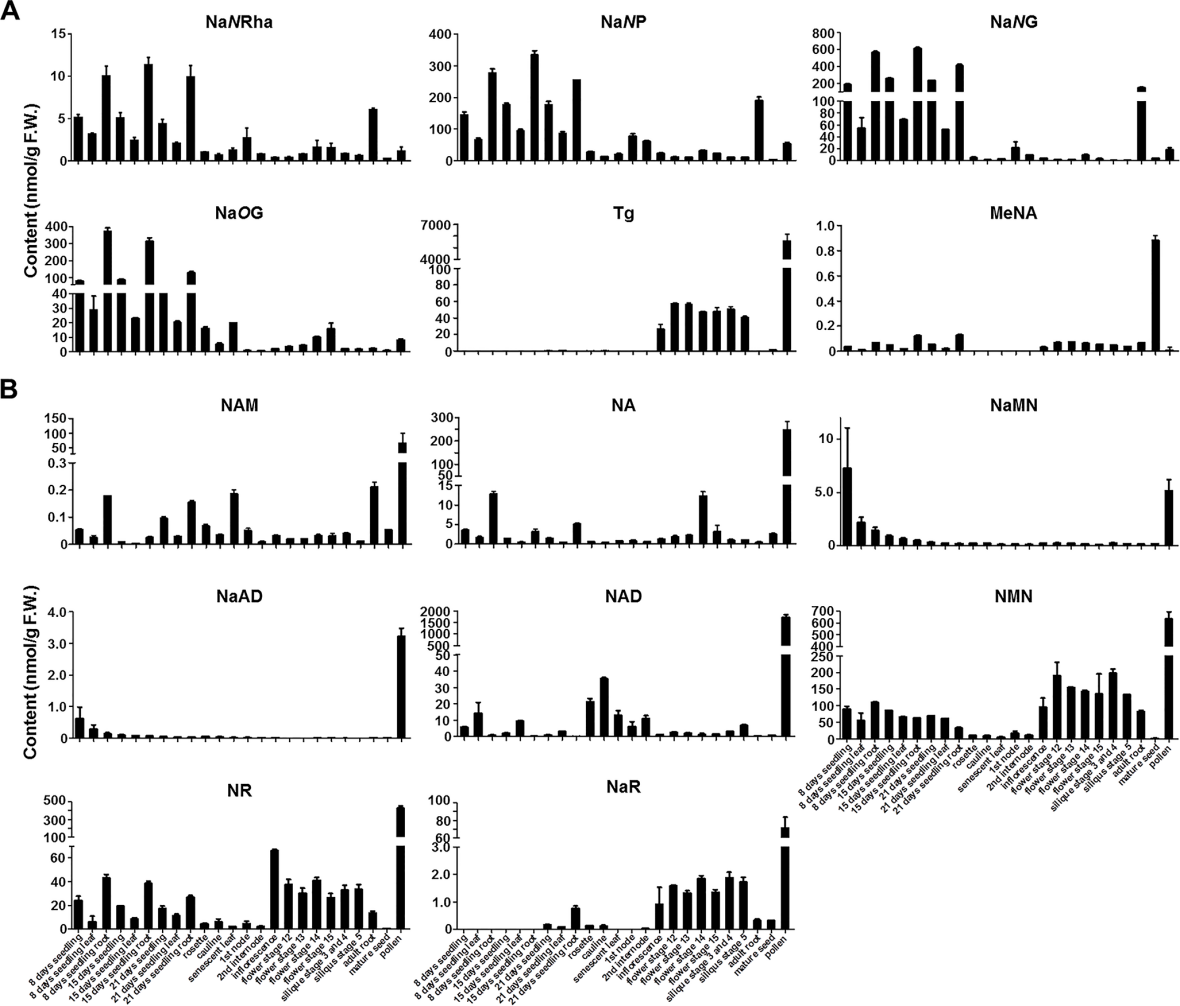
Measurement of endogenous NA conjugates **(A)** and NAD-related compounds **(B)** in 24 different tissues of *Arabidopsis*. It is noteworthy that MeNA was measured using different methods (detailed see Wu et al., 2018). F.W., fresh weight. The abbreviations for chemical name see **Figure 1** legend. Bars show means ± SDs (*n* = 3). F.W., fresh weight. Other abbreviations: Na*N*Rha, nicotinate *N*-rhamnoside; Na*N*P, nicotinate *N*-pentoside; NaR, nicotinate riboside; NMN, nicotinamide mononucleotide; NR, nicotinamide riboside.

As it is hard to separate these two novel NA glycosides from Na*N*G using radio-TLC as we performed previously, we thus develop a targeted LC-QQQ-MS method with optimized MRM mode (**Supplemental Table 1**) to quantity these two chemicals and other NAD-related chemicals in 24 different tissues of *Arabidopsis thaliana* (detailed sample information see **Supplemental Table 3**). The analytical results showed that both two novel NA *N*-glycosides were highly accumulated in root tissues, from either young seedlings or adult plants (**Figure 3A**). This pattern is similar to Na*N*G, which suggested that the genes responsible for the production of novel NA *N*-glycosides is highly expressed in *Arabidopsis* roots.

There are three other intriguing results from the chemical analysis. First, NMN is highly accumulated in all tested tissues except the mature seeds, although the physiological function is unclear thus far. NMN could be re-utilized for NAD biosynthesis catalyzed by NaMNAT (encoded by *At5g55810*), which adenylates both NMN and NaMN *in vitro* (Hashida et al., 2007). Whether NR (nicotinamide riboside) and NaR could be utilized for NAD production is also an open question to be further clarified. Second, trigonelline content was extremely high in pollen when compared with other tissues (**Figure 3A**). However, the gene encoding trigonelline synthase (also called nicotinate *N*-methyltransferase, At3g53140) was barely expressed in pollen (Li et al., 2017), which suggested that a transporter protein might be involved in trigonelline accumulation in pollen. Previously, a NA/trigonelline transporter protein (encoded by At3g13050) had been characterized by using a comparative genomics strategy (Jeanguenin et al., 2012). Interestingly, *At3g13050* was predominantly expressed in mature pollen based on the data from TAIR website (https://www.arabidopsis.org/; **Supplemental Figure 2**; (Winter et al., 2007)). Third, the chemicals involved in NAD salvage pathway, including NAM, NA, NaMN, NaAD, and NAD, are predominantly accumulated in mature pollens (**Figure 3B**). This phenomenon is consistent with a previous report (Hashida et al., 2013). High-NAD accumulation in dry pollen and fast decrease in germinated pollen suggested that NAD biosynthetic pathway (*de novo* and salvage pathway) plays an important role in pollen germination and pollen tube elongation. Further experiments are needed to reveal the mechanism of this phenomenon.

### UGT76C5 Is a Multiple Functional Nicotinate *N*-Glycosyltransferase in *Arabidopsis*

**Figure 4:**
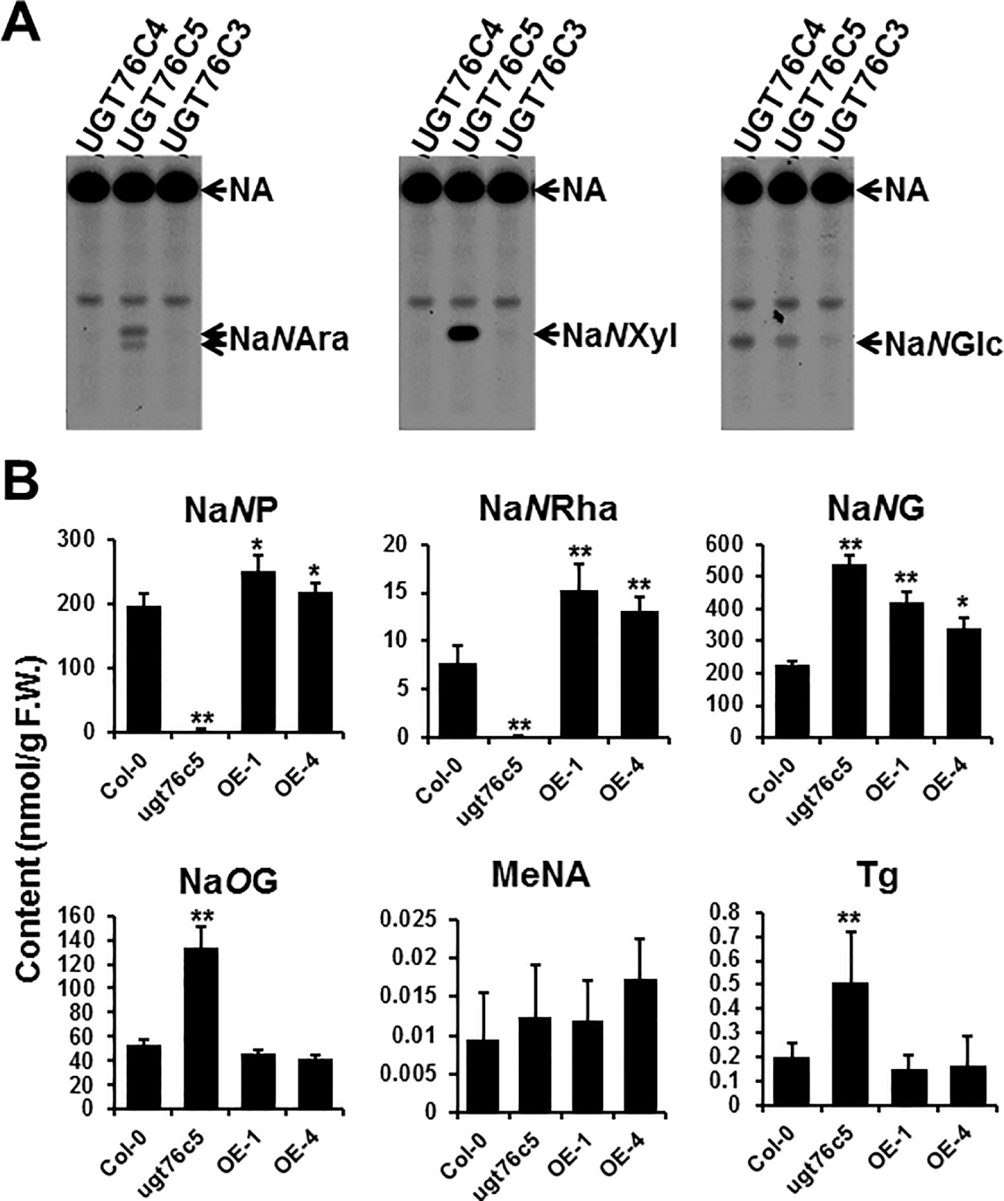
Characterization of UGT76C5 in *Arabidopsis thaliana*. **(A)** Glycosyltransferase assays using purified recombinant UGT76C proteins incubated with [^14^C]-NA and UDP-arabinose, UDP-xylose, or UDP-glucose as substrates. **(B)** Chemical analysis of the 10-day-old whole seedlings of *UGT76C5* transgenic plants. It is noteworthy that MeNA was measured using different method (detailed see Wu et al., 2018). Bars show means ± SDs (*n* = 3). Asterisks indicate significant differences from the Col-0 plants; *P < 0.05, **P < 0.01 (two-tailed Student’s *t* test). F.W., fresh weight.

Previously, we had identified UGT76C4 and UGT76C5 as NA *N*-glucosyltransferase; both UGT76C4 and UGT76C5 also showed root-predominant expression pattern. The promiscuous property of plant small molecule glycosyltransferase led us to test whether UGT76C4 and UGT76C5 are responsible for NA pentosylation and/or rhamnosylation in *Arabidopsis*. UGT76C3 (encoded by *At5g05900*), which is homologous to UGT76C4 and UGT76C5, without a known function thus far, was also included in the NA glycosyltransferase assays using [^14^C]-NA and UDP-arabinose or UDP-xylose as co-substrate. The assays showed that UGT76C5, rather than UGT76C3 and UGT76C4, catalyzed the NA pentosylation with a high efficiency. Consistent with previous results, both UGT76C4 and UGT76C5 could utilize UDP-glucose and NA as substrate (**Figure 4A**). We did not test rhamnosyltransferase activity since UDP-rhamnose is not commercially available. To the best of our knowledge, UGT76C5 is the first multiple functional *N*-glycosyltransferase identified from plant species. Other UDP-sugars, e.g., UDP-Gal (UDP-galactose), UDP-GlcA (UDP-glucuronic acid), and UDP-GlcNA (UDP-*N*-acetylglucosamine), are not included in this study, as both UGT76C4 and UGT76C5 showed little activity toward these sugar donors (Li et al., 2015).

Using the above-mentioned LC-QQQ-MS method, we further analyzed the knock-out mutants and overexpression lines of *UGT76C5* (all overexpression lines are in Col-0 genetic background), which had been characterized in our previous study (Li et al., 2015). The results showed that the content of Na*N*P and Na*N*Rha are significantly decreased in *76c5* mutant (**Figure 4B**). Detection of low abundance of Na*N*P and Na*N*Rha also suggested that other glycosyltransferases might also catalyze the NA *N*-pentosylation or NA *N*-rhamnosylation. It is noteworthy that Na*N*P and Na*N*Rha did not increase too much in UGT76C5 overexpressor plants when compared with wild type. In contrast, overexpression of *UGT76C4* leads to around 200-fold higher Na*N*G content than wild-type plants (Li et al., 2015). One possible explanation is that the limited availability of UDP-xylose and UDP-arabinose, rather than UDP-glucose, in *Arabidopsis* prevents the accumulation of Na*N*P and Na*N*Rha. Moreover, Na*N*G content was increased by two-fold in the *ugt76c5* mutant compared to that of WT. In a biochemist’s view, Na*N*P or/and Na*N*Rha may repress UGT76C4’s (Na*N*GT) activity in *Arabidopsis* and remove of Na*N*P and Na*N*Rha in *ugt76c5* mutant increase the UGT76C4’s activity to some extent.

We also tested whether Na*N*P or/and Na*N*Rha could be secreted from plants, mainly from root tissue. The results clearly showed that all nicotinate conjugates, except MeNA, can be detected in the liquid medium (**Supplemental Figure 3**). Consistent with the chemical profiling in planta (**Figure 4B**), little Na*N*P and Na*N*Rha were found in the liquid medium cultured with *ugt76c5* mutant (**Supplemental Figure 3**).

### One Amino Acid Determines the Sugar Donor Specificities of *Arabidopsis* Nicotinate *N*-Glycosyltransferases

**Figure 5:**
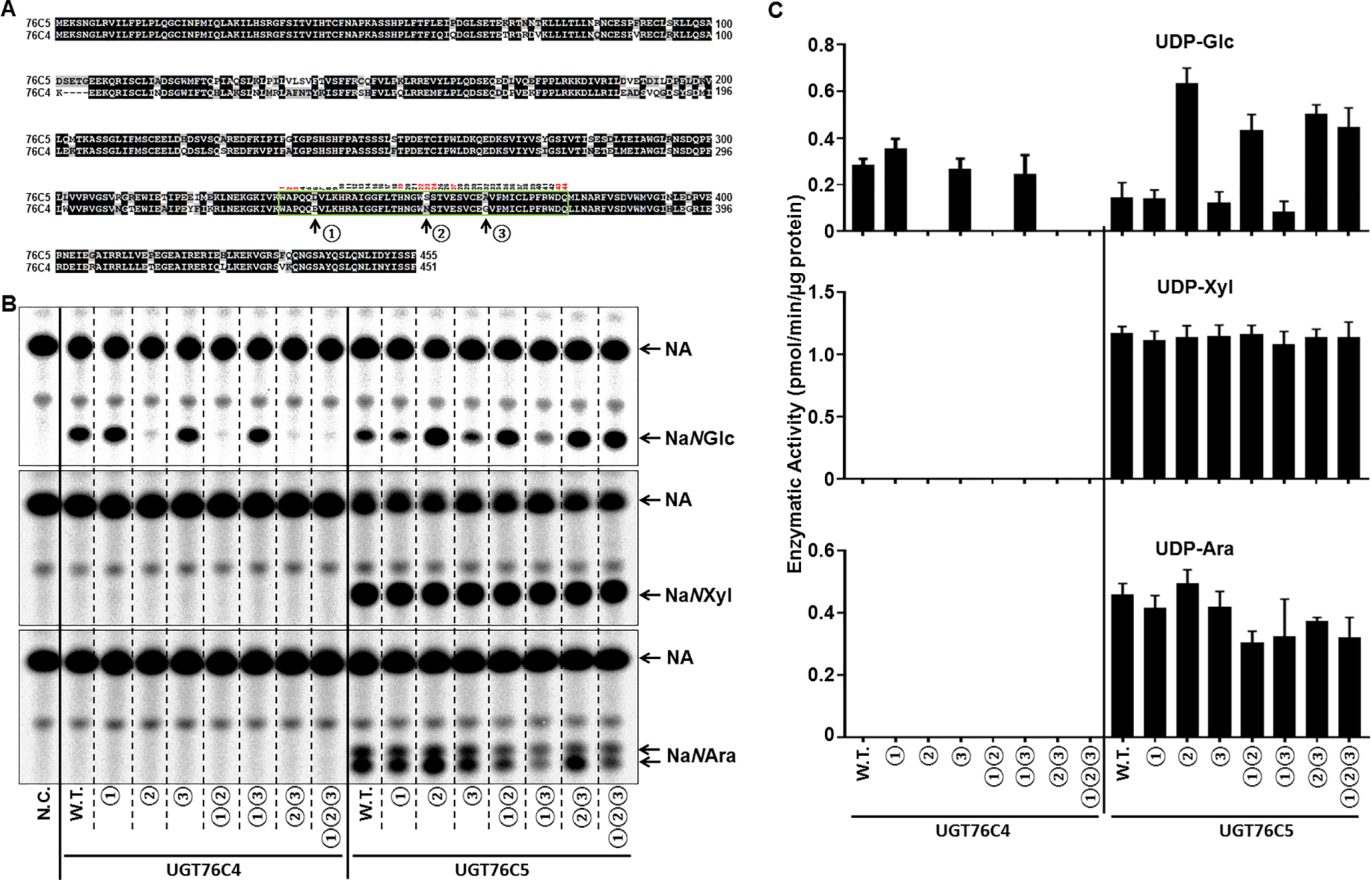
Glycosyltransferase assay of UGT76C4, UGT76C5, and their mutants. **(A)** Protein sequence alignment of UGT76C4 and UGT76C5. The PSPG motif was boxed in green, and the different amino acids in PSPG motif between UGT76C4 and UGT76C5 were indicated with black arrows and numbers ① (D340 in UGT76C5 or E336 in UGT76C4), ② (S357 in UGT76C5 or N353 in UGT76C4), and ③ (A366 in UGT76C5 or G362 in UGT76C4). Among 44 amino acids in PSPG motif, the amino acids marked with red number are putatively bound to UDP-sugars. **(B)** Biochemical assay of UGT76C4, UGT76C5, and their mutants with different sugar donors and [^14^C]-NA. N.C., negative control; only purified MBP tag protein was added in this reaction. W.T., wild-type. ①, amino acid interconversion between D and E; ②, amino acid interconversion between S and N; and ③, amino acid interconversion between A and G. **(C)** Comparisons of glycosyltransferase activity of UGT76C4 and UGT76C5 wild-type and mutant enzymes with various UDP-sugars. W.T., wild type. Bars show means ± SDs (*n* = 3).

Both UGT76C4 and UGT76C5 shared 79.6% identity on protein level and recognized NA as sugar acceptor. The difference in sugar donor specificity between UGT76C4 (UDP-glucose) and UGT76C5 (UDP-glucose, UDP-xylose, and UDP-arabinose, and probably also UDP-rhamnose) prompted us to further study the mechanism underlying this phenomenon. PSPG motif (total 44 amino acids in almost all identified family 1 GTs) is highly conserved in all glycosyltransferases involved in a plant specialized metabolism, as this motif is thought to correspond to a UDP-binding site (Caputi et al., 2012). Variations in PSPG motif had been demonstrated to play an important role in the UDP-sugar donor specificity of plant family 1 GTs (Noguchi et al., 2009; Ono et al., 2010; Zong et al., 2019). There are three amino acids difference in the PSPG motif of UGT76C4 and UGT76C5: position 6 (D in UGT76C5 *vs*. E in UGT76C4), 23 (S in UGT76C5 *vs*. N in UGT76C4), and 32 (A in UGT76C5 and G in UGT76C4) in a total 44 amino acids in the common (**Figure 5A**). In order to further study the sugar donor specificity of UGT76C4 and UGT76C5, the different residues in PSPG motif were interconverted between these two nicotinate *N*-glycosyltransferases (**Figure 5A**). The UGT76C4 mutant N23S (the amino acids are re-numbered in PSPG motif) completely lost the NA *N*-glucosyltransferases activity (**Figure 5B**). Substitution of E6 (E6D) and G32 (G32A) showed a slight change in the Na*N*GT activity: E6D mutant had a slight increase (25%; *n* = 3), while G32A had a slight decrease (6%; *n* = 3) compared to wild-type UGT76C4 activity. It is noteworthy that S23N mutation in UGT76C5 led to around five-fold increase Na*N*GT activity, and D6E and A32G mutants had little effect on the Na*N*GT activity of UGT76C5 (**Figures 4B, C** and **Table 1**). These results reveal that N23 in PSPG motif plays a critical role for Na*N*GT activity. Comprehensive sequence analysis of Family 1 GTs showed that N23 is highly conserved in all tested GT proteins with the exception of group-A GTs, in which N23 was replaced with G (glycine) or S (serine). Altogether, N23 is important for the maximal catalytic efficiency of glucosyltransfer activity, whereas G or S at the same position is probably required for recognition of other sugar donors (Caputi et al., 2012), like the Na*N*PT in this study. Meanwhile, all mutants of UGT76C5 did not significantly change in NA *N*-pentosyltransferase activity, using either UDP-arabinose or UDP-xylose as sugar donor (**Figure 5C**).

**Table 1:** Catalytic efficiency of wild-type UGT76C5 and mutants. Kinetic parameters for UDP-glucose were determined with 1.0 mM NA.

Enzyme	*K* *_m_* (mM)	*K* *_cat_* (x 10^-3^, s^-1^)	*K* *_cat_*/*K* *_m_* (s^-1^M^-1^)
UGT76C5 (WT)	0.33 ± 0.022	0.89 ± 0.053	2.69 ± 0.11
76C5②	0.17 ± 0.008	2.04 ± 0.053	12.0 ± 0.26
76C5①②	0.20 ± 0.007	1.55 ± .072	7.70 ± 0.17
76C5②③	0.19 ± 0.019	1.56 ± 0.11	8.36 ± 0.31
76C5①②③	0.21 ± 0.009	1.49 ± 0.033	6.93 ± 0.17

Moreover, other residues located outside the PSPG motif also played important role the recognition of UDP-sugars (Osmani et al., 2008; Ono et al., 2010; Louveau et al., 2018). Further experiments, e.g., crystal structural comparison of UGT76C4 and UGT76C5, are needed to elucidate the mechanism difference between UGT76C4 and UGT76C5.

In summary, we first performed the comprehensive NAD metabolomics across 24 tissues of *Arabidopsis thaliana*, which provided fundamental knowledge for studying the physiological functions of NA conjugates and the NAD salvage pathway. UGT76C5 is the first multiple functional *N*-glycosyltransferase with sugar-donor promiscuity characterized from plant species. One amino acid in PSPG motif is identified for sugar-donor specificity of UGT76C4 and UGT76C5. These results increase our understanding of the relationship between NAD biosynthesis and NA conjugations and should also allow for additional experiments to provide insights into NAD homeostasis when plants response and adapt to (a)biotic stresses. Two recent studies demonstrated that the plant specialized metabolites (both focus on terpenoids) played a key role to modulate the root microbiota (Chen et al., 2019; Huang et al., 2019). The data and materials generated in this study pave the way for further understanding of the physiological functions of NA conjugates in plants, e.g., whether these root-specific NA glycosides involved into root microbiota modulation in *Arabidopsis thaliana*.

## Data Availability Statement

The datasets generated for this study are available on request to the corresponding author.

## Author Contributions

LL and GL performed the molecular and biochemical experiments and analyzed the data. FZ carried out LC-QQQ-MS analysis and analyzed the data. GW conceived the project, supervised the experiments, and completed the writing.

## Funding

This work was financially supported by the National Key Research and Development Projects (2018YFA0900600), the “Priority Research Program” of the Chinese Academy of Science (ZDRW-ZS-2019-2-0202), and the State Key Laboratory of Plant Genomics of China (SKLPG2016A-13 and SKLPG2016B-13) to G.W.

## Conflict of Interest

The authors declare that the research was conducted in the absence of any commercial or financial relationships that could be construed as a potential conflict of interest.
