# Corrigendum: Qualitative and Quantitative NAD^+^ Metabolomics Lead to Discovery of Multiple Functional Nicotinate *N*-Glycosyltransferase In *Arabidopsis*

**DOI:** 10.3389/fpls.2019.01648

**Published:** 2020-01-08

**Authors:** Lingyun Liu, Fengxia Zhang, Guosheng Li, Guodong Wang

**Affiliations:** ^1^ State Key Laboratory of Plant Genomics and National Center for Plant Gene Research (Beijing), Institute of Genetics and Developmental Biology, The Innovative Academy of Seed Design, Chinese Academy of Sciences, Beijing, China; ^2^ College of Advanced Agricultural Sciences, University of the Chinese Academy of Sciences, Beijing, China

**Keywords:** NAD, nicotinate, *N*-glycosyltransferase, Preiss-Handler pathway, *Arabidopsis*

There is an error in the **Funding** statement. The correct number for **“**National Key Research and Development Projects**”** is **“**2018YFA0900600.**”** The corrected **Funding** statement appears below.

“This work was financially supported by the National Key Research and Development Projects (2018YFA0900600), the “Priority Research Program” of the Chinese Academy of Science (ZDRW-ZS-2019-2-0202), and the State Key Laboratory of Plant Genomics of China (SKLPG2016A-13 and SKLPG2016B-13) to G.W.”

Additionally, in the original article, there was a mistake in **Table 1** as published. The unit for Kcat/Km should be “s^-1^M^-1^” instead of “x10^-3^, s^-1^M^-1^”. The corrected **Table 1** appears below.

The authors apologize for this error and state that this does not change the scientific conclusions of the article in any way. The original article has been updated.

**Table 1:** Catalytic efficiency of wild-type UGT76C5 and mutants. Kinetic parameters for UDP-glucose were determined with 1.0 mM NA.

Enzyme	*K_m_* (mM)	*K_cat_* (x 10^-3^, s^-1^)	*K_cat_*/*K_m_* (s^-1^M^-1^)
UGT76C5 (WT)	0.33 ± 0.022	0.89 ± 0.053	2.69 ± 0.11
76C5②	0.17 ± 0.008	2.04 ± 0.053	12.0 ± 0.26
76C5①②	0.20 ± 0.007	1.55 ± .072	7.70 ± 0.17
76C5②③	0.19 ± 0.019	1.56 ± 0.11	8.36 ± 0.31
76C5①②③	0.21 ± 0.009	1.49 ± 0.033	6.93 ± 0.17

