# Supplementary material for: Qualitative and Quantitative NAD+ Metabolomics Lead to Discovery of Multiple Functional Nicotinate N-Glycosyltransferase in Arabidopsis
Source: Front Plant Sci. 2019 Sep 27;10:1164. doi: 10.3389/fpls.2019.01164 (PMC6776627; doi:10.3389/fpls.2019.01164)
Supplement: Supplementary file 1 [file Image_1.pdf]

SUPPLEMENTAL DATA

Supplemental Table 1. The MRM Settings and Retention Time for Metabolites Analyzed in This Study.

Metabolite	RT (min) [a]	Prec Ion	Prod Ion	Dwell	Frag (V)	CE (V)	Cell Acc (V)	Polarity
**NaAD**	8.53	665	136	30	380	35	3	Positive
**NAD**	8.61	664	136	30	380	37	3	Positive
**NaMN**	8.64	336	124	30	380	13	3	Positive
**NMN**	8.81	335	123	30	380	8	3	Positive
**NA*O*G**	3.78	286	124	30	380	30	3	Positive
**NA*N*G**	8.57	286	124	30	380	11	3	Positive
**NaR**	8.15	256	124	30	380	9	3	Positive
**NR**	8.21	255	123	30	380	9	3	Positive
**NA**	5.79	124	80	30	380	20	3	Positive
**NAM**	1.21	123	80	30	380	13	3	Positive
**MeNA**	1.43 [b]	138	52	30	380	50	3	Positive
**Tg**	7.48	138	92	30	380	22	3	Positive
**Na*N*P**	8.42	256	124	30	380	9	3	Positive
**Na*N*Rha**	8.25	270	124	30	380	12	3	positive

[a] Prec Ion: Precursor Ion, Prod Ion: Product Ion, Frag: Fragmentor Voltage, CE: Collision Energy, Cell Acc: Cell Accelerator Voltage, RT: Retention Time.

[b] The retention time of MeNA was obtained with different LC-MS program (see Wu et al., 2018).

Supplemental Table 2. Primers Used in This Study.

Primer Name	Sequence information (5' to 3')[1]	Function
76C5FOR	GGAATTCATGGAGAAGAGTAATGGCCTTCGAGTGA (EcoR I site was underlined)	Protein expression by pMal-c2x
76C5REV	GCTCTAGACTAAAAAGATGATATATAATCAATCAAATTTTGTAACGATTG (Xba I site was underlined)	
76C5F1_site mut	TGGGCTCCGCAACAAGA**G**GTTCTAAAGCATCGAGCCATTGG	Site-directed mutagenesis of 76C5
76C5R1_site mut	TCGATGCTTTAGAAC**C**TCTTGTTGCGGAGCCCATTTCAC	
76C5F2_site mut	CACATAATGGTTGGA**A**CTCGACTGTTGAGAGTGTTTGTGAAGGC	Site-directed mutagenesis of 76C5
76C5R2_site mut	CTCTCAACAGTCGAG**T**TCCAACCATTATGTGTCAGGAATCCC	
76C5F3_site mut	AGAGTGTTTGTGAAG**GC**GTCCCTATGATCTGTTTGCCTTTTC	Site-directed mutagenesis of 76C5
76C5R3_site mut	ACAGATCATAGGGAC**GC**CTTCACAAACACTCTCAACAGTCGAG	
76C4FOR	GGAATTCATGGAGAAGAGTAATGGCCTGCGAGT (EcoR I site was underlined)	Protein expression by pMal-c2x
76C4REV	GCTCTAGACTAGAAAGATGATATATAATTAATCAAATTTTGTAGAGATTGATATG (Xba I site was underlined)	
76C4F1_site mut	GGGCTCCACAACAAGA**C**GTTCTAAAGCATCGAGCTATTGGAGG	Site-directed mutagenesis of 76C4
76C4R1_site mut	TCGATGCTTTAGAAC**G**TCTTGTTGTGGAGCCCATTTCACTATCT	
76C4F2_site mut	CACATAATGGTTGGA**G**CTCGACGGTTGAGAGTGTTTGTGA	Site-directed mutagenesis of 76C4
76C4R2_site mut	CACTCTCAACCGTCGAG**C**TCCAACCATTATGTGTCAAGAAACCT	
76C4F3_site mut	AGAGTGTTTGTGAAG**CA**GTCCCTATGATCTGTTTGCCTTTTCG	Site-directed mutagenesis of 76C4
76C4R3_site mut	ACAGATCATAGGGAC**TG**CTTCACAAACACTCTCAACCGTCG	

[1] mutated nucleotide and corresponding amino acid codon were highlighted.

Supplemental Table 3. List of 24 tissues used for NAD metabolomics analysis in this study.

Sample name	Tissue	Age	Growth Condition
Sample_1	Young Seedlings	8 days	½ MS, long day (16/8)
Sample_2	Young seedling leaf	8 days	½ MS, long day (16/8)
Sample_3	Young seedling roots	8 days	½ MS, long day (16/8)
Sample_4	Young Seedlings	15 days	½ MS, long day (16/8)
Sample_5	Young seedling leaf	15 days	½ MS, long day (16/8)
Sample_6	Young seedling roots	15 days	½ MS, long day (16/8)
Sample_7	Young Seedlings	21 days	½ MS, long day (16/8)
Sample_8	Young seedling leaf	21 days	½ MS, long day (16/8)
Sample_9	Young seedling roots	21 days	½ MS, long day (16/8)
Sample_10	Rosette leaf	30 days	Soil, long day (16/8)
Sample_11	Cauline leaf	30 days	Soil, long day (16/8)
Sample_12	Senescent leaves	35 days	Soil, long day (16/8)
Sample_13	1st node	35 days	Soil, long day (16/8)
Sample_14	Stem, 2nd internode	35 days	Soil, long day (16/8)
Sample_15	Inflorescence	35 days	Soil, long day (16/8)
Sample_16	Flowers stage 12	30 days	Soil, long day (16/8)
Sample_17	Flowers stage 13	30 days	Soil, long day (16/8)
Sample_18	Flowers stage 14	30 days	Soil, long day (16/8)
Sample_19	Flowers stage 15	30 days	Soil, long day (16/8)
Sample_20	Siliques, with seeds stage 3 and 4	30 days	Soil, long day (16/8)
Sample_21	Siliques, with seeds stage 5	30 days	Soil, long day (16/8)
Sample_22	Adult roots	35 days	Soil, long day (16/8)
Sample_23	Mature seeds	42 days	Soil, long day (16/8)
Sample_24	Fresh harvested pollen	30 days	Soil, long day (16/8)

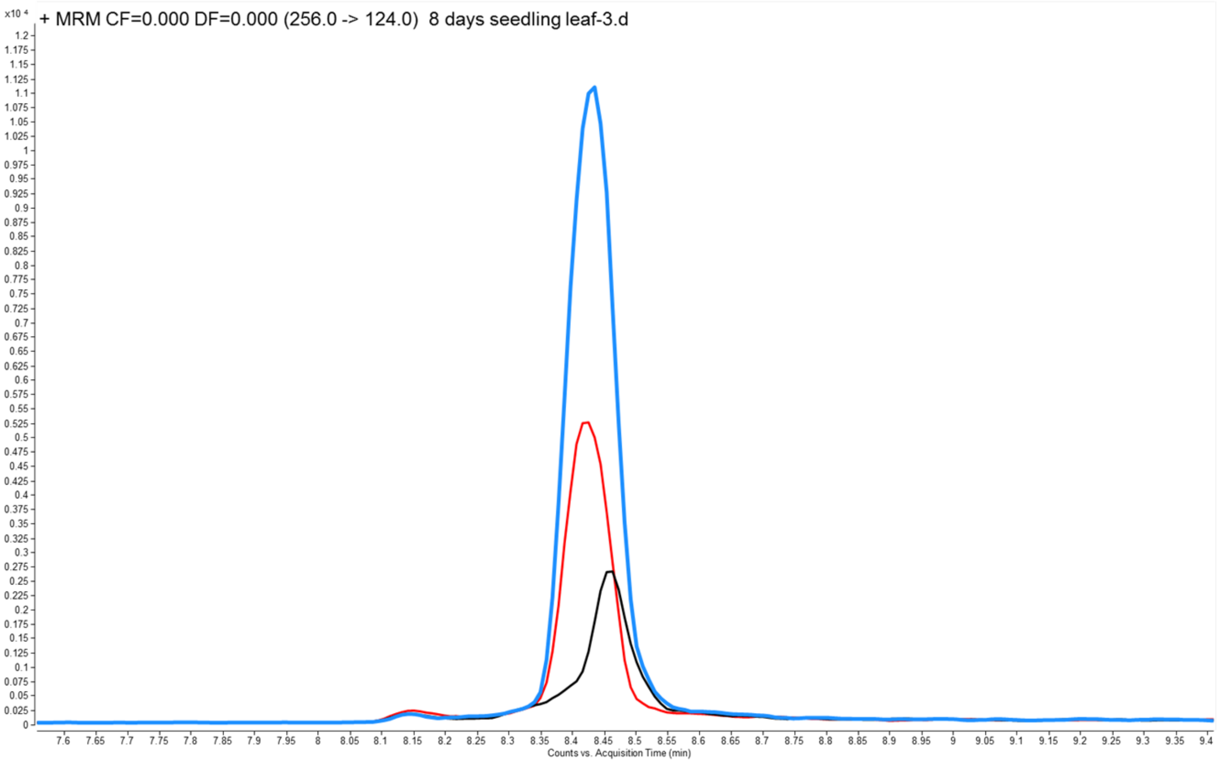

Supplemental Figure 1. LC-QQQ-MS analysis (MRM, *m/z* 256 > 124) of nicotinate *N*-arabinoside (black line), nicotinate *N*-xyloside (red line) and the extract from leaf tissue of 8-day-old seedlings (blue line). Both nicotinate *N*-arabinoside and nicotinate *N*-xyloside are prepared by enzymatic reactions catalyzed by UGT76C5.

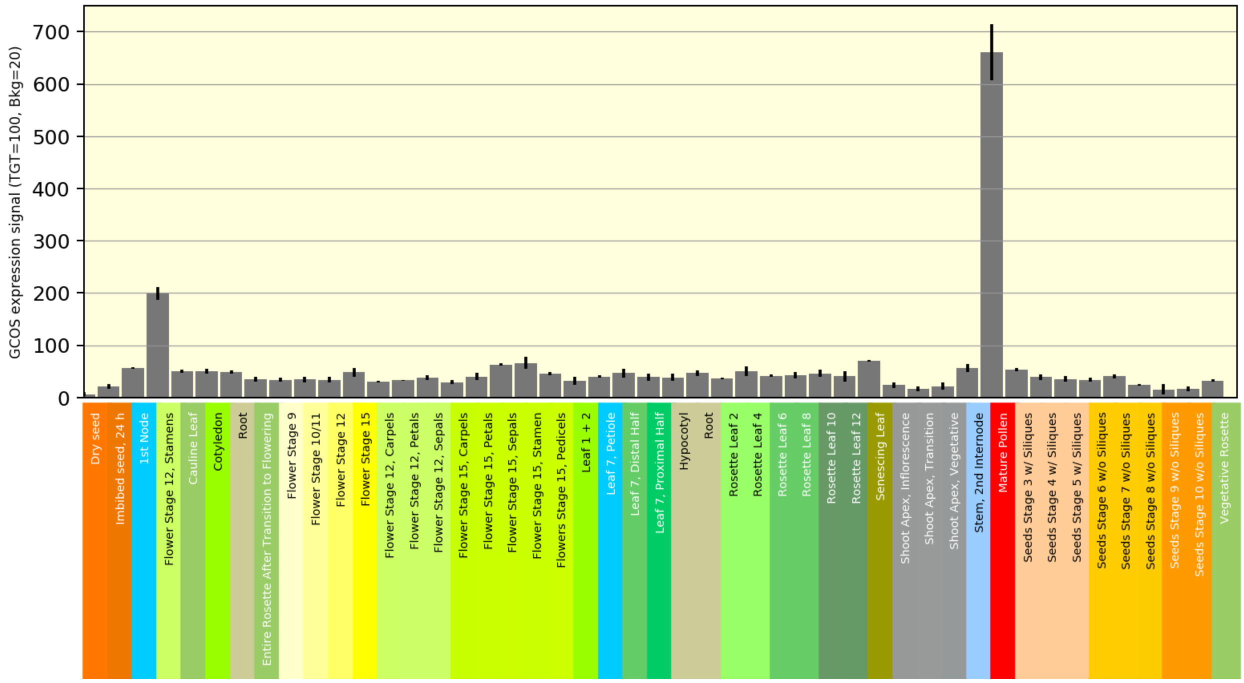

Supplemental Figure 2. Expression pattern of *At3g13050* in Arabidopsis, which was extracted from TAIR website (https://www.arabidopsis.org/; Winter et al., 2007).

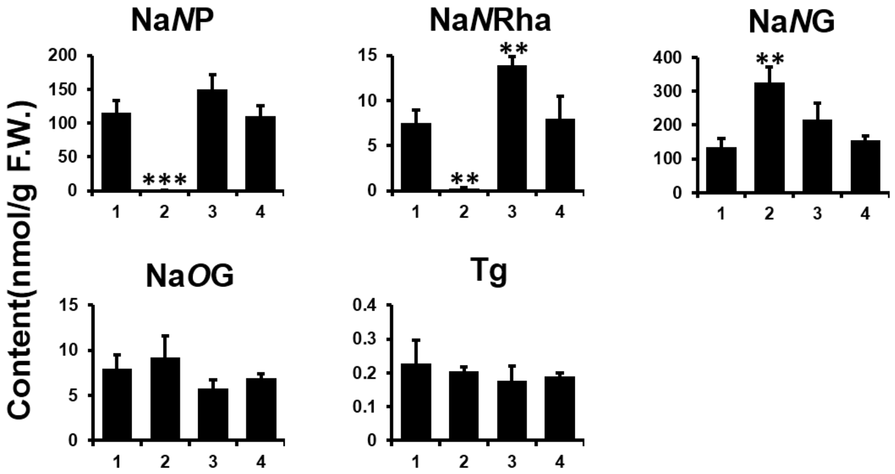

Supplemental Figure 3. Quantification of nicotinate conjugates secreted in the liquid medium. In these experiments, around 3 mg of seeds, after sterilized with 75% alcohol and washed with ddH_2O five times, were grown in 20 mL ½ MS liquid medium in a growth chamber for 15 days. The plant materials were weighed and 500 µL of liquid medium was mixed with equal volume of acetonitrile. The liquid samples were injected on UPLC-MS/MS for analysis after 10 minutes of maximum speed centrifugation with a microcentrifuge and filtered through a 0.22-µm syringe filter. Bars shown mean ± SD (n = 3). Asterisks indicate significant differences from the Col-0 plants; *** $P < 0.001$, ** $P < 0.01$ (two-tailed Student's t test). F.W., fresh weight.

REFERENCES

Winter D, Vinegar B, Nahal H, Ammar R, Wilson GV, Provart NJ (2007) An "electronic fluorescent pictograph" browser for exploring and analyzing large-scale biological data sets. *PLoS One* 2, e718. doi: 10.1371/journal.pone.0000718

Wu, R.R., Zhang, F.X., Liu, L.Y., Li, W., Pichersky, E., and Wang, G.D. (2018). MeNA, controlled by reversible methylation of nicotinate, is an NAD precursor that undergoes long-distance transport in Arabidopsis. *Mol. Plant* 11, 1264-1277. doi: 10.1016/j.molp.2018.07.003
